# Bronchiectasis as a Comorbidity of Chronic Obstructive Pulmonary Disease: A Systematic Review and Meta-Analysis

**DOI:** 10.1371/journal.pone.0150532

**Published:** 2016-03-15

**Authors:** Qingxia Du, Jianmin Jin, Xiaofang Liu, Yongchang Sun

**Affiliations:** 1 Department of Respiratory Medicine and Department of Emergency, Beijing Tongren Hospital, Capital Medical University, Beijing, 100730, China; 2 Department of Respiratory and Critical Care Medicine, Peking University Third Hospital, Beijing, 100191 China; University at Buffalo, State University of New York, UNITED STATES

## Abstract

**Background:**

Bronchiectasis revealed by chest computed tomography in COPD patients and its comorbid effect on prognosis have not been addressed by large-sized studies. Understanding the presence of bronchiectasis in COPD is important for future intervention and preventing disease progression.

**Methods:**

Observational studies were identified from electronic literature searches in Cochrane library, PubMed, ScienceDirect databases, American Thoracic Society and European Respiratory Society meeting abstracts. A systematic review and meta-analysis of studies was performed to summarize the factors associated with bronchiectasis in COPD patients. Primary outcomes included the risks for exacerbation frequency, isolation of a potentially pathogenic microorganism, severe airway obstruction and mortality. Odds ratios (ORs) were pooled by random effects models.

**Results:**

Fourteen observational studies were eligible for the study. Compared with COPD without bronchiectasis, comorbid bronchiectasis in COPD increased the risk of exacerbation (1.97, 95% CI, 1.29–3.00), isolation of a potentially pathogenic microorganism (4.11, 95%CI, 2.16–7.82), severe airway obstruction (1.31, 95% CI, 1.09–1.58) and mortality (1.96, 95% CI, 1.04–3.70).

**Conclusions:**

The presence of bronchiectasis in patients with COPD was associated with exacerbation frequency, isolation of a potentially pathogenic microorganism, severe airway obstruction and mortality.

## Introduction

Chronic obstructive pulmonary disease (COPD) is one of the leading causes of morbidity and mortality around the world [1]. It is a complex and heterogeneous disease; the pathological and structural abnormalities, and clinical features vary greatly among patients despite having a similar lung function [2–3]. Identifying patient groups with unique specific characteristics and clinical consequences is needed to guide therapy and management of COPD, especially for those at high risk for exacerbation and mortality, in whom a specific intervention could be tested [4].

Thoracic computed tomography is currently a noninvasive imaging tool that holds promise for phenotyping in COPD, as it reveals significant differences in morphological changes in the lungs even with similar degree of airflow limitation [5]. Underlying structural changes in COPD, such as bronchiectasis, may also modulate exacerbation severity and contribute to morbidity associated with exacerbations [6]. In 2014, the Global Initiative for Chronic Obstructive Lung Disease (GOLD) firstly describes bronchiectasis as one of the comorbidities of COPD [7]. Bronchiectasis and COPD share common characteristics in clinical presentations and pathophysiology such as chronic cough, sputum production, susceptibility to recurrent exacerbations and incompletely reversible airflow obstruction [8].

Several studies [9–11] have revealed an association between bronchiectasis and COPD, reporting that the presence of bronchiectasis in patients with COPD is associated with increased bronchial inflammation, frequent colonization of airway by potentially pathogenic microorganisms (PPMs) and severe airflow obstruction. However, another observational study [12] suggests that the number of exacerbations and bacterial colonization are not related to bronchiectasis in COPD patients.

Given the potentially important impact of coexistent bronchiectasis on COPD outcomes, we summarized the current data from observational cohort studies to investigate the association between bronchiectasis and COPD.

## Methods

### Literature Search Strategy and Study Identification

We followed the recommendations of the Preferred Reporting Items for Systematic Reviews and Meta-Analyses (PRISMA) statement [13] to perform this systematic review. Two investigators independently and in duplicate conducted a systematic review of the literature search of Cochrane library, PubMed, ScienceDirect databases, and abstracts from American Thoracic Society and European Respiratory Society, as well as traced citations through July 30, 2015 to obtain all relevant published literature. Disease-specific search terms “COPD” and “chronic obstructive lung disease” were combined with “bronchiectasis” in all our searches. All subject headings and abstracts were examined.

The inclusion criteria are as follows: (1) clearly defined bronchiectasis as a primary comorbidity or in a subgroup of patients with COPD in the study; (2) the presence of bronchiectasis was assessed by chest computed tomography; (3) presented odds ratio (OR), hazard ratio (HR) and corresponding confidence interval (CI) for the presence of bronchiectasis on outcomes, or gave enough data to calculate these parameters. Studies are excluded: (1) if the presence of bronchiectasis was assessed by chest X-ray only; (2) if the study only included occupational and single sex subjects.

### Primary Outcomes

The outcomes are risks of COPD exacerbations, isolation of a PPM, severe airflow obstruction and overall mortality. Objective evidence of COPD exacerbations includes reporting an increase in at least two out of three clinical symptoms, hospital admission for a COPD exacerbation, emergency room visits, and prescription of antibiotic or corticosteroid courses. Objective evidence of a PPM includes positive sputum cultures for *Haemophilus influenzae*, *Streptococcus pneumoniae*, *Moraxella catarrhalis*, *Haemophilus parainfluenzae*, *Staphylococcus aureus*, *Pseudomonas aeruginosa*, *Klebsiella pneumoniae*, and other gram-negative bacteria[14–15].

### Data Abstraction and Quality Assessment

Two investigators (YS and QD) independently extracted the data. The extraction information was as follows: the first authors’ names, study locations, publication dates, follow-up periods, baseline characteristics of study populations, diagnostic methods of bronchiectasis, outcome of interest and covariates. The quality of cohort studies was evaluated by Newcastle-Ottawa Scale (available at: http://www.ohri.ca/programs/clinical_epidemiology/oxford.asp).

### Data Synthesis and Analysis

The Stata version 12.0 SE (Stata Corp.) was used to calculate pooled effect sizes and 95% CIs. Statistical heterogeneity across studies was quantified using the I^2^ statistic, and I^2^≧50% was considered a substantial level of heterogeneity[15]. As the study populations are substantially different, random effects model was used and individual study characteristics was explored.

Publication biases were evaluated by visually examining the symmetry of funnel plots [16], and P < 0.1 indicates significant asymmetry. We also performed the Duval and Tweedie nonparametric “trim and fill” procedure to further assess the possible effect of publication bias [17].

## Results

### Description of Included Studies

**Fig 1:**
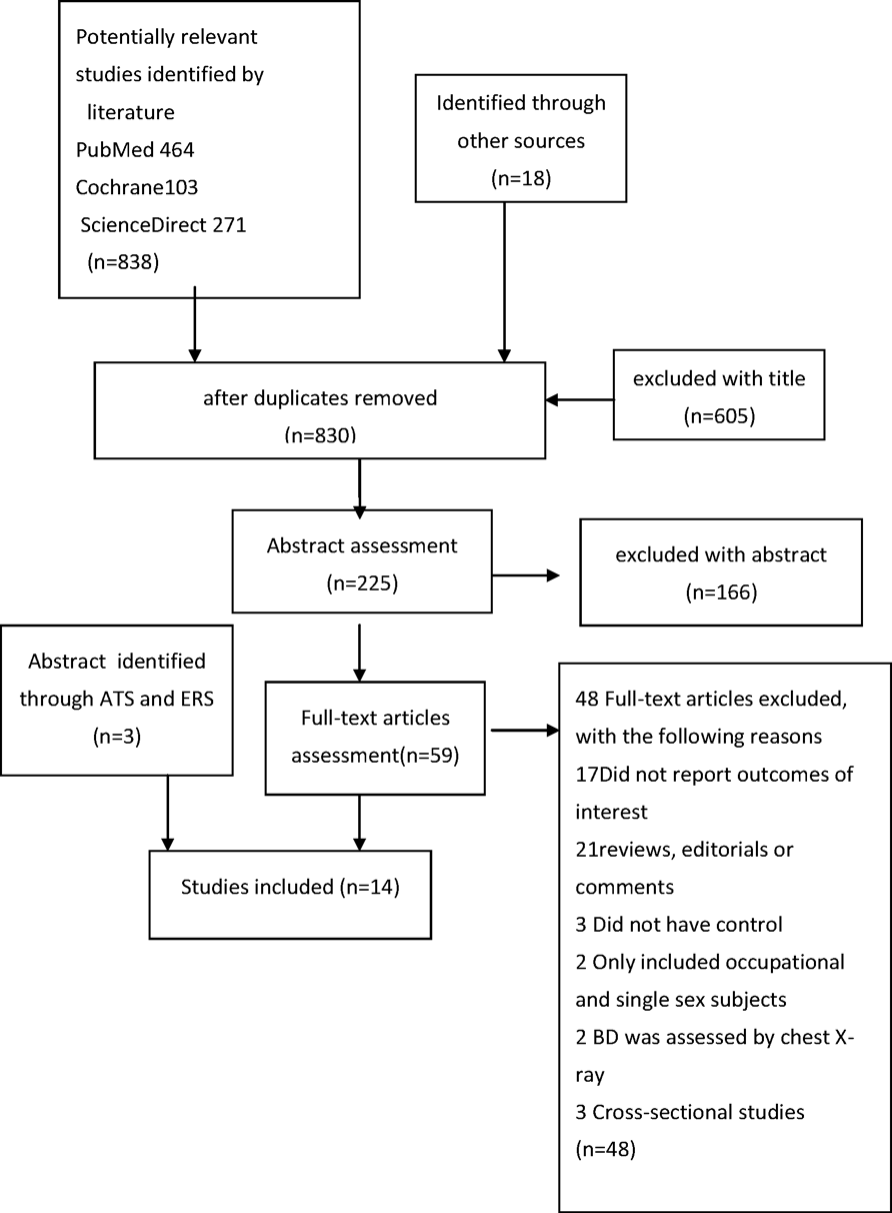
Flowchart of process of literature review and selection.

The process of selection of studies is shown in Fig 1. The study characteristics and findings are summarized in Table 1. A total of 14 studies met the inclusion criteria, including 5329 COPD patients, and1572 (29.5%) patients were comorbid with bronchiectasis. Association of the presence of bronchiectasis with COPD exacerbations (ECOPD) [9–12,18, 19,20]was reported in seven studies, isolation of PPMs[9,10,12,21,22]and specifically of *Pseudomonas aeruginosa* (*P*. *aeruginosa*) [9,10,23,24]in five and four studies respectively, severe airflow obstruction[9,11, 12, 18,19,25,26] in seven studies, mortality in four studies[10, 19,23,27], and higher levels of inflammatory cytokines in three studies[10,11,21]which were not enough to conduct a meta-analysis.

**Table 1:** Characteristics of Included Studies in the Meta-analysis.

Author/year/country	Duration	Number (bronchiectasis/total COPD)	Participants	Diagnostic methods(bronchiectasis /PPM)	Outcomes (Odd ratios)	Adjustments/considerations
Katsura.H[27]/2001/ Japan	five years	unknown /157	Age mean 79.2, severe COPD on long-term domiciliary oxygen therapy	-	Mortality 3.96(1.39–11.28).	-
Patel.I.S.[21]/2004/United Kingdom	1,197 (804–1,941) days	27/54	Moderate and severe COPD in East London COPD Study.	HRCT, by Smith/ Quantitative culture	PPM(7.0,1.47–37.9); longer symptom recovery time at exacerbation (p < 0.001).Higher levels of airway inflammatory cytokines.	-
Roche N [22]/2006/ France	Two years	23/118	Age 68.4±12.1, consecutive hospitalized for ECOPD.	HRCT / Quantitative culture	Positive quantitative culture (2.61, 1.09–6.26).	-
Garcia-Vidal.C[25]/2009/Spain	One year	46/88	Age 72.11±10.0, consecutive hospitalized for ECOPD.	HRCT	Severe airflow obstruction (1.27, 0.99–1.64).	-
Bafadhel M[26]/2011/ England	mean 4.35 years	20/75	Age mean 69.1, recruited consecutively from respiratory clinics	CT scans	Severe airflow obstruction (0.97, 0.93–1.02).	-
Martinez-Garcia MA[12]/2011 /Spain	2004–2006	53/92	Age 71.3+9.3, moderate and severe COPD	HRCT/by Naidich/ quantitative culture	Severe airflow obstruction (3.87, 1.38–10.5); PPM (3.59, 1.3–9.9); ECOPD (3.07, 1.07–8.77).	Gender, age, smoking, treatment, MRC dyspnea, daily sputum.
Stewart JI[18]/2012/ United States	2009–2011	758/3636	65.5 ± 8.1 vs. 62.8 ± 8.6 years	CT	Severe airflow obstructive (1.13, 1.06–1.25); ECOPD (1.04,1.01–1.3).	-
Eman O. Arram[9]/ 2012/ Egypt	median 23 months	33/69	Age 67±9, moderate and severe COPD.	HRCT/ Sputum cultures	Severe airflow obstructive (3.61, 1.33–9.83); PPM (2.42, 0.09–6.46); *P*.*Aeruginosa* (18.57, 5.54–62.30); ECOPD (4.75, 1.67–13.52).	-
Martine-Garcia MA[10] /2013/ Spain	median 48 months	115/201	Age 70.3±8.9, moderate and severe COPD.	HRCT / by Naidich	Mortality (2.54, 1.16–5.56); ECOPD(2.34,1.36–4.01); PPM(7.69,2.87–20.56); higher CRP(P = 0.018) *P*.*Aeruginosa*(3.08, 0.98–9.6).	Age, post-bronchodilator FEV1% value, MRC dyspnea,PO2, body mass index, presence of PPM in sputum, presence of daily sputum production, number of severe exacerbations, Charlson Index, and peripheral albumin and ultrasensitive CRP concentration.
Tulek B[11]/2013/ Turkey	two years	27/80	Age 68±8, out-patients COPD	HRCT/ by modified Bhalla scoring system	Severe airflow obstructive (1.77,1.26–2.53); higher CRP levels;ECOPD (2.08,1.20–3.60);	
Timothy Gatheral[23]/2014/ United Kingdom	3.5(0.9–6.6) years.	278/406	Age 71±11, admitted with first exacerbation.	CT scans	*P*.*Aeruginosa*(1.39, 1.07–1.80), mortality HR (1.05, 0.95–1.17); Inpatient days (p < 0.001)	Age, gender, Charlson Index, and increasing severity emphysema and bronchial wall thickening.
Gallego M[24]/2014/ Spain	over one year 1003 ± 306 days	56/118	Age 69.5 ± 8.2, a post bronchodilator FEV1 below 50%.	HRCT / by Smith.	*P*. *aeruginosa* (OR 9.8, 95% CI: 1.7 to 54.8)	Age, smoking history, FEV1, body mass index, BODE score, co-morbidities, influenza and pneumococcal vaccination and long-term oxygen therapy use.
Sadigov AS[19]/ 2014/ Azerbaijan	Cohort/ Median 12 months	26/54	Consecutive patients with severe and very severe COPD	HRCT/-	Severe airflow obstructive (1.77,1.0–3.12);Mortality (2.15,1.28–3.59); ECOPD(2.2,1.31–3.7).	Unadjusted
Jairam PM [20]/2015/ Netherlands	case–cohort study / median 4.4 years	110/338	routine chest CT scanning for non-pulmonary indications	Routine diagnostic chest CT/ pulmonary lobe-based visual grading system	ECOPD(1.5,0.9–2.5)	-

CI = confidence interval; CRP = C-reaction protein; ECOPD = exacerbation of COPD; HR = hazard ratio; HRCT = High-resolution computed tomography; OR = odds ratio; *P*. *aeruginosa* = *Pseudomonas aeruginosa*; PPM = potentially pathogenic microorganism.

### Quality of Included Studies

All these studies were observational cohorts, and thus there were limitations in the interpretation of causality and in methodologies. The sample size ranged from 54 to 3636, and seven studies had a small population (N<100), while three studies were published in abstracts [18, 19, 27]. One study was conducted in the United States, 2 in the United Kingdom, 4 in Spain, 1 in France, 1 in Egypt, 1 in Azerbaijan, and 1 in Turkey. Follow-up varied from one year [24] to five years [27]. Most studies lacked adjustment for potential confounders. In seven articles, bronchiectasis was graded by scoring systems proposed by Smith [21, 24], Naidich [10, 12] and Bhalla [11], and other systems [18, 20] respectively. Selection of subjects was either from primary care, outpatient recruitment, or from hospitalization. The main outcomes of the studies are described as follows. The quality of each study, assessed by the Newcastle-Ottawa Scale, was displayed in Table A in S1 File (median score, 8.5; range, 7 to 9.).

### Bronchiectasis Exposure and Risk for COPD exacerbations

**Fig 2:**
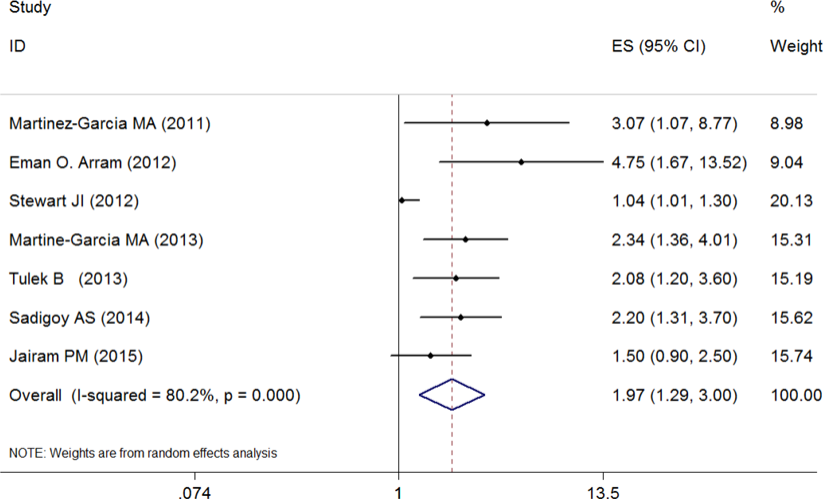
Odd ratios for the association between comorbid bronchiectasis and risk for COPD exacerbations.

Fig 2 presents ORs (with 95% CIs) for all seven studies assessing the association between bronchiectasis exposure and risk for COPD exacerbations. The pooled ORs of 1.97 (95% CI, 1.29–3.00) indicated that comorbid bronchiectasis was associated with COPD exacerbations. There was a high heterogeneity among studies (I^2^ = 80.2%, P<0.01).Two studies [21, 23] reported longer time to recovery from exacerbation in COPD patients with bronchiectasis.

### Bronchiectasis Exposure and Risk for Isolation of Potentially Pathogenic Microorganisms

**Fig 3:**
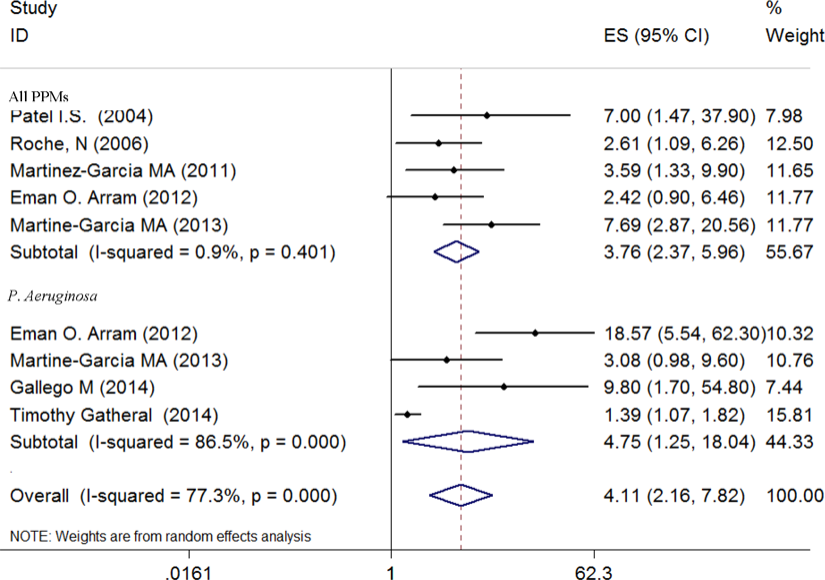
Odd ratios for the association between comorbid bronchiectasis and risk for isolation of a potentially pathogenic microorganism.

Fig 3 presents ORs (with 95% CIs) for all five studies assessing the risk for isolation of a PPM and four studies specifically on *P*. *aeruginosa*. The pooled ORs for a PPM of 3.76 (95% CI, 2.37–5.96) and for *P*. *aeruginosa* of 4.75 (95% CI, 1.25–18.04) indicated that the presence of bronchiectasis was associated with increased risk for isolation of PPMs in the airways. There was no evidence of statistical heterogeneity of ORs for all PPMs across studies (P = 0.401; I^2^ = 0.9%), but significant heterogeneity among studies reporting isolation of *P*. *aeruginosa* (I^2^ = 86.5%, P<0.01).

### Bronchiectasis Exposure and Risk for Severe Airway Obstruction

**Fig 4:**
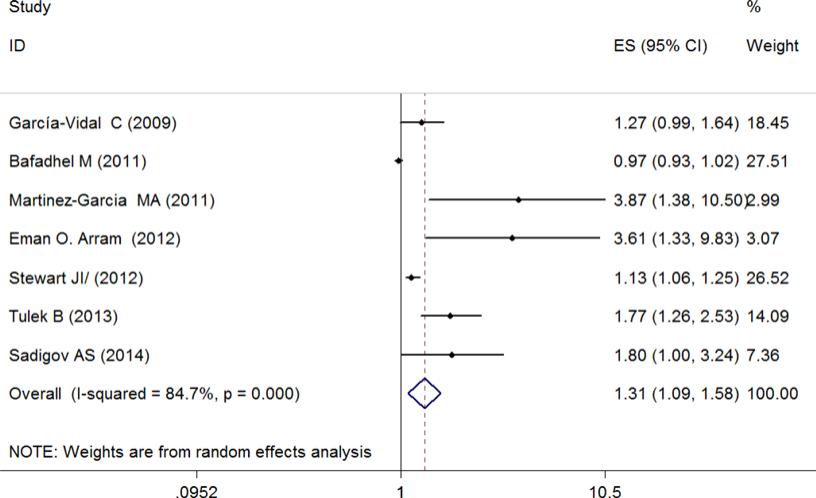
Odd ratios for the association between comorbid bronchiectasis and risk for severe airway obstruction.

Fig 4 presents ORs (with 95% CIs) for all seven studies assessing the association between the presence of bronchiectasis and risk for severe airway obstruction. The pooled ORs of 1.31 (95% CI, 1.09–1.58) indicated that the presence of bronchiectasis was associated with increased risk for severe airway obstruction. Significant between-studies heterogeneity was evident and the I^2^ (P<0.01) was 84.7%, indicating that 84.7% of the observed variance came from real differences between the studies.

### Bronchiectasis Exposure and Risk for Mortality

**Fig 5:**
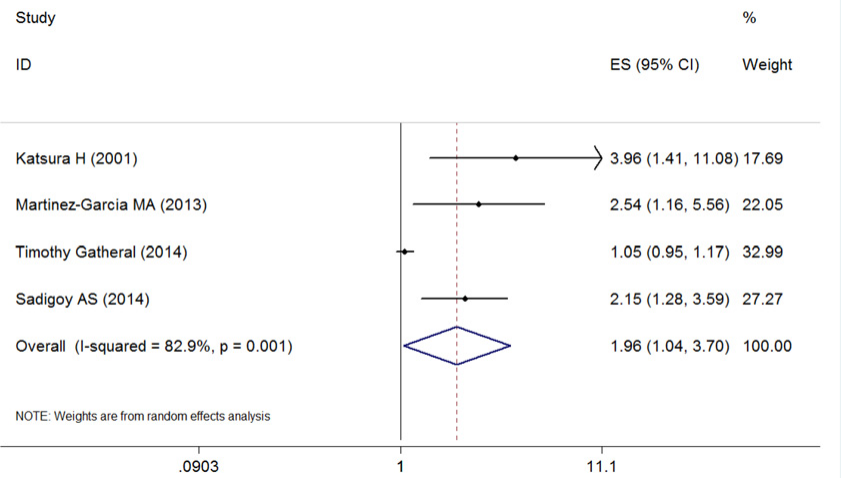
Odd ratios for the association between comorbid bronchiectasis and risk for mortality.

Fig 5 presents ORs (with 95% CIs) for all four studies assessing the association between the presence of bronchiectasis and mortality. The pooled ORs of 1.96 (95% CI, 1.04–3.70) indicated that the presence of bronchiectasis was associated with increased risk for mortality. Only one study [23] reported no correlation between the presence of bronchiectasis and survival, but in this study the authors showed that patients with bronchiectasis had a shorter survival following CT scan (2.6 (0.5–6.7) years) than those with no evidence of bronchiectasis (3.8(1.4–6.6) years) (p = 0.046). Significant between-studies heterogeneity and substantial variance were evident (I^2^ = 82.9%, P = 0.001).

There was high heterogeneity among all studies, which was mostly due to clinical heterogeneity of the various study settings and populations. In addition, funnel plots were produced and yielded significant evidence of publication bias. The Begg’s tests were also statistically significant (P<0.00). We undertook sensitivity analysis using the trim and fill method, which conservatively imputed hypothetical negative unpublished studies to mirror the positive studies and produced a symmetrical funnel plot (Figs A, B, C,D in S1 File) [17], and the pooled analysis incorporating the hypothetical studies continued to show a statistically significant association between bronchiectasis and outcomes.

## Discussion

In this meta-analysis, we found that bronchiectasis revealed by chest CT was common in COPD patients, and these patients were more likely to have exacerbations or a longer recovery time from exacerbations. They also had a higher risk for isolation of a PPM including *P*. *aeruginosa* from sputum, and for more severe airflow obstruction than subjects without bronchiectasis. Finally, COPD patients with bronchiectasis may have a higher mortality risk. Therefore, the presence of bronchiectasis is no mere radiological findings but has a real impact on the natural history of COPD itself and could be of prognostic value in the evaluation and management of the disease.

Exacerbations of COPD are important events in the course of the disease because they have a negative impact on a patient’s quality of life, accelerate lung function decline, and are associated with morbidity and mortality [7]. The most common cause of exacerbations of COPD is believed to be respiratory tract infection [7]. However, the cause about one-third of severe exacerbations of COPD cannot be identified [7]. One study showed that some patients were particularly prone to exacerbations, and were defined as frequent exacerbators[28]. The reasons why the presence of bronchiectasis in COPD can be related to more frequent and longer duration of exacerbations are speculative. Recurrent COPD exacerbations are associated with the presence of heightened airway bacterial and inflammatory burden [29, 30] and worsening of GOLD spirometric grades [28, 31]. In this meta-analysis, we found that the presence of bronchiectasis in COPD patients was also related to isolation of PPMs and poor lung function. Chronic bacterial infection, especially by *P*. *aeruginosa*, was associated with more advanced disease and mortality in both COPD and bronchiectasis patients [32–35]. The presence of bacteria in the lower airways in COPD impairs host defense mechanisms, which results in epithelial cell integrity disruption and inflammation, further airway structural damage, which could be the mechanism for longer and more severe COPD exacerbations [19]. Incomplete resolution of bacterial infection or bacterial colonization was considered to be a risk for relapse, and therefore appropriate and effective antibiotic therapy for bacterial infection in exacerbation of COPD with bronchiectasis may be important in preventing exacerbations and relapses or delaying subsequent exacerbations [36–38]. But to date, appropriate modalities for bacterial eradication, particularly for those with chronic bacterial colonization, haven’t been established.

The association between the presence of bronchiectasis and COPD mortality is a remarkable finding of our study. This could be explained by the potential impact of brochiectasis on COPD. Firstly, the presence of bronchiectasis is associated with an increased risk for exacerbations. It is well known that exacerbations accelerate the rate of decline of lung function and are associated with significant mortality. Secondly, bronchiectasis is associated with severe airflow obstruction, which is also a risk for COPD death [28, 31]. Thirdly, bronchiectasis is associated with systemic and pulmonary inflammation, which may be the mechanistic link between COPD and comorbid conditions, such as cardiovascular diseases, lung cancer and others which are also contributors to mortality [39].

The cause and effect relationship between bronchiectasis and COPD is still controversial. It is possible that chronic infection by bacteria in the airways of COPD may form a vicious cycle of structural damage, loss of epithelial cell integrity, impaired mucociliary clearance, and mucus hyper-secretion which results in further mucosal injury and inflammation [40–42]. Persistent airway inflammation with ensuing tissue injury and remodeling may induce bronchiectasis.

Our study has a number of strengths. Firstly, to the best of our knowledge, the present meta-analysis provides the most comprehensive data about the association between bronchiectasis in COPD and clinical outcomes. One of recent meta-analysis [43] on this topic was performed on limited studies (larger studies such as the COPDgene CT results were not included) and had not enough data to draw conclusion on mortality. Furthermore, they included a study of selected COPD patients with bronchial wall thickening rather than evident bronchiectasis [44]. Secondly, we followed the PRISMA statement guidelines [13] to identify, screen and describe the protocols in this systematic review, including a very sensitive systematic literature search strategy, an independent and duplicate selection and data abstraction processes, and a strict evaluation of study quality. Finally, to minimize the possibility of publication bias, we included three high-quality abstracts data from American Thoracic Society and European Respiratory Society.

It also needs to be mentioned that this meta-analysis has several limitations. Firstly, it was not possible to grade COPD by lung function in all the studies, and therefore, the precise prevalence of bronchiectasis in different stages of COPD cannot be calculated. Secondly, the selected publications are of varying definitions and quality with regards to defining the severity of COPD and the specific CT findings (particularly extent and severity of bronchiectasis) for each patient. Only five of the studies used a scoring tool to measure the degree of bronchiectasis, but none of these studies described a testing phase to show that the scoring tool was fit-for-purpose in their populations. This makes pooling of the data somewhat problematic and perhaps explains some of the heterogeneity noted among studies. Thirdly, although the primary objective of this study was to assess the effects of bronchiectasis on COPD clinical outcomes, the available data from these studies provide evidence for an association but do not establish causality. Lastly, there are limitations in the methodological quality of the included studies. No prospective large cohort study was found; the included studies were relatively small in size, and most of the patients were with moderate to severe airway obstruction.

Despite these, the available data are still sufficient for us to perform a reliable meta-analysis. Our results support that coexistent bronchiectasis in patients with COPD is associated with worse respiratory symptoms, higher risk for exacerbations and mortality. For improving symptoms and reducing future risks, this subgroup of patients may need more directed therapies targeting chronic mucus production and airway bacterial infection in addition to recommended treatments for COPD.

## Conclusions

### Implications for clinical practice

This study adds to the fast growing evidence of the association between bronchiectasis and poor outcomes in COPD. It has important implications for our current practice and approach to the evaluation and management of COPD, because COPD patients with comorbid bronchiectasis may benefit from specific therapies.

### Implications for Research

Well designed prospective studies are clearly needed to explore the clinical significance of detection of bronchiectasis by HRCT in the management of COPD. Further higher-quality and longer-term studies are also needed to address the causal relationship between bronchiectasis and COPD clinical outcomes, as well as the therapeutic benefit of targeting bronchietasis (such as antibiotics) in COPD. Clearly, the pathophysiological mechanisms underlying the coexistence of bronchiectasis and COPD, and the candidate genes associated with the bronchiectasis phenotype of COPD are awaiting further studies.

## Supporting Information

S1 File Table A, The Quality of Observational Studies Assessed by Newcastle–Ottawa Scale. Table B, List of the 48 full-text excluded articles. Fig A, Funnel plots of publication bias for comorbid bronchiectasis and risk for COPD exacerbations. Fig B, Funnel plots of publication bias for comorbid bronchiectasis and risk for isolation of PPMs and P. Aeruginosa. Fig C, Funnel plots of publication bias for comorbid bronchiectasis and risk for airway severe obstruction. Fig D, Funnel plots of publication bias for comorbid bronchiectasis and risk for mortality. Appendix 1, Commands for each figure.
(PDF)

S2 File PRISMA Checklist. (DOC)

S3 File Primary data for each figure. (XLSX)
