# Supplementary material for: Bronchiectasis as a Comorbidity of Chronic Obstructive Pulmonary Disease: A Systematic Review and Meta-Analysis
Source: PLoS One. 2016 Mar 15;11(3):e0150532. doi: 10.1371/journal.pone.0150532 (PMC4792534; doi:10.1371/journal.pone.0150532)
Supplement: S1 File — Table A, The Quality of Observational Studies Assessed by Newcastle–Ottawa Scale. Table B, List of the 48 full-text excluded articles. Fig A, Funnel plots of publication bias for comorbid bronchiectasis and risk for COPD exacerbations. Fig B, Funnel plots of publication bias for comorbid bronchiectasis and risk for isolation of PPMs and P. Aeruginosa. Fig C, Funnel plots of publication bias for comorbid bronchiectasis and risk for airway severe obstruction. Fig D, Funnel plots of publication bias for comorbid bronchiectasis and risk for mortality. Appendix 1, Commands for each figure. (PDF) [file pone.0150532.s001.pdf]

Bronchiectasis as a Comorbidity of Chronic Obstructive Pulmonary Disease

A Systematic Review and Meta-analysis

Qingxia Du, Jianmin Jin, Xiaofang Liu,Yongchang Sun

Table A. The Quality of Observational Studies Assessed by Newcastle – Ottawa Scale

		Newcastle –Ottawa Scale									
		Selection				Comparability		Outcome			Total stars
Study	Year	1	2	3	4	5A	5B	6	7	8	94
Gompertz S [13]	2001	B(*)	A(*)	A(*)	A(*)	A(*)	NO	A(*)	A(*)	A(*)	8
Katsura H [14]	2001	-	-	-	-	-	-	-	-	-	-
Pate l I.S.[15]	2004	NO	A(*)	A (*)	A(*)	A(*)	B(*)	A(*)	A(*)	A(*)	8
Roche N [16]	2006	B(*)	A(*)	A (*)	A(*)	A(*)	B(*)	A(*)	A(*)	A(*)	9
García-Vidal C[17]	2009	B(*)	A(*)	A (*)	A(*)	A(*)	B(*)	A(*)	A(*)	A(*)	9
Bafadhel M [18]	2011	B(*)	A(*)	A (*)	A(*)	A(*)	NO	A(*)	A(*)	A(*)	8
Martinez-Garcia MA[19]	2011	B(*)	A(*)	A (*)	A(*)	A(*)	B(*)	A(*)	A(*)	A(*)	9
Eman O. Arram[20]	2012	B(*)	A(*)	A (*)	A(*)	A(*)	B(*)	A(*)	A(*)	NO	8
Martine-Garcia MA [21]	2013	B(*)	A(*)	A (*)	A(*)	A(*)	B(*)	A(*)	A(*)	A(*)	9
Tulek B [22]	2013	B(*)	A(*)	A (*)	A(*)	A(*)	NO	A(*)	A(*)	A(*)	8
Timothy Gatheral[23]	2014	B(*)	A(*)	A (*)	A(*)	A(*)	B(*)	A(*)	A(*)	A(*)	9
Gallego M [24]	2014	B(*)	A(*)	A (*)	A(*)	A(*)	B(*)	A(*)	A(*)	A(*)	9

Table B. The list of 48 full-text excluded articles.

No	Title	Exclusion reasons
1	Siafakas N M, Vermeire P, Pride N B, et al. Optimal assessment and management of chronic obstructive pulmonary disease (COPD)[J]. European Respiratory Journal, 1995, 8(8): 1398-1420.	Review
2	Keistinen T, Saynajakangas O, Tuuponen T, et al. Bronchiectasis: an orphan disease with a poorly-understood prognosis[J]. European Respiratory Journal, 1997, 10(12): 2784-2787.	Review
3	Jones A P, Rowe B H. Bronchopulmonary hygiene physical therapy for chronic obstructive pulmonary disease and bronchiectasis[J]. The Cochrane Library, 1998.	Review
4	O'Brien C, Guest PJ, Hill SL, Stockley RA. Physiological and radiological characterisation of patients diagnosed with chronic obstructive pulmonary disease in primary care. *Thorax*. 2000;55(8):635–642.	cross-sectional study

5	Jones A, Rowe B H. Bronchopulmonary hygiene physical therapy in bronchiectasis and chronic obstructive pulmonary disease: a systematic review[J]. Heart & Lung: The Journal of Acute and Critical Care, 2000, 29(2): 125-135.	Review
6	Gompertz S, Bayley D L, Hill S L, et al. Relationship between airway inflammation and the frequency of exacerbations in patients with smoking related COPD. *Thorax.* 2001; 56:36–41	Did not have control
7	Wilkinson T M, Patel I S, Wilks M, et al. Airway bacterial load and FEV1 decline in patients with chronic obstructive pulmonary disease.[J]. American Journal of Respiratory & Critical Care Medicine, 2003, 167(8):1090-1095.	No outcomes of interest
8	Altin R, Savranlar A, Kart L, et al. Presence and HRCT quantification of bronchiectasis in coal workers[J]. European Journal of Radiology, 2004, 52(2):157-163.	Occupational subjects
9	Antoni R, Eduard M, Néstor S, et al. Microbiologic determinants of exacerbation in chronic obstructive pulmonary disease.[J]. Archives of Internal Medicine, 2005, 165(8):891-897.	No outcomes of interest
10	Gursel G. Determinants of the length of mechanical ventilation in patients with COPD in the intensive care unit[J]. Respiration, 2005, 72(1): 61-67.	No outcomes of interest
11	Hoffman E A, Simon B A, Geoffrey M L. State of the Art. A structural and functional assessment of the lung via multidetector-row computed tomography: phenotyping chronic obstructive pulmonary disease.[J]. Proceedings of the American Thoracic Society, 2006, 3(6):519-32.	Review
12	Fujimoto K, Kitaguchi Y, Kubo K, Honda T. Clinical analysis of chronic obstructive pulmonary disease phenotypes classified using high-resolution computed tomography. *Respirology*. 2006;11(6):731–740.	No outcomes of interest
13	Gursel G. Does coexistence with bronchiectasis influence intensive care unit outcome in patients with chronic obstructive pulmonary disease?[J]. Heart & Lung the Journal of Critical Care, 2006, 35(1):58-65.	cross-sectional study/ bronchiectasis was confirmed by Chest X-ray
14	Martínez-García M A, Perpiñá-Tordera M, Soler-Cataluña J J, et al. Dissociation of lung function, dyspnea ratings and pulmonary extension in bronchiectasis.[J]. Respiratory Medicine, 2007, 101(11):2248–2253.	Review
15	Wedzicha J A, Hurst J R. Structural and functional co-conspirators in chronic obstructive pulmonary disease exacerbations.[J]. Proceedings of the American Thoracic Society, 2007, 4(8):602-605.	Review

16	Ley-Zaporozhan J, Ley S, Kauczor H U. Morphological and functional imaging in COPD with CT and MRI: present and future.[J]. European Radiology, 2008, 18(3):510-521.	Review
17	Chang A, Bilton D. Exacerbations in cystic fibrosis: 4--Non-cystic fibrosis bronchiectasis.[J]. Thorax, 2008, 63(3):269-276.	No outcomes of interest
18	Chang A B, Grimwood K, Maguire G, et al. Management of bronchiectasis and chronic suppurative lung disease in indigenous children and adults from rural and remote Australian communities[J]. The Medical journal of Australia, 2008, 189(7): 386-93.	No outcomes of interest
19	Han MK, Bartholmai B, Liu LX, et al. Clinical significance of radiologic characterizations in COPD. *COPD*. 2009;6(6):459–467.	No outcomes of interest
20	Kim W, Oh Y J, Lee Y, et al. CT scanning-based phenotypes vary with ADRB2 polymorphisms in chronic obstructive pulmonary disease.[J]. Respiratory Medicine, 2009, 103(1):98-103.	No outcomes of interest
21	Moncelly L , Maurer C , Roche N , et al. [Acute COPD exacerbations in women: EABPCO-CPHG study by the College of general hospital pneumologists].[J]. Revue De Pneumologie Clinique, 2010, 66(2):107-19.	Single sex subjects
22	Agusti A. Prognosis of COPD patients requiring frequent hospitalization: role of airway infection.[J]. Respiratory Medicine, 2010, 104(6):840–848.	Review
23	Agusti A, Calverley P, Celli B, Coxson HO, Edwards LD, Lomas D, MacNee W, Miller BE, Rennard S, Silverman EK, Tal-Singer R, Wouters E, Yates JC, Vestbo J. Characterisation of COPD heterogeneity in the ECLIPSE cohort. Respir Res. 2010;11:122.	cross-sectional study
24	Mair G, Maclay J, Miller J J, et al. Airway dimensions in COPD: relationships with clinical variables.[J]. Respir Med, 2010, 104(11):1683–1690.	Do not have control
25	Valderrey A D, Pozuelo M J, Jiménez P A, et al. Chronic colonization by Pseudomonas aeruginosa of patients with obstructive lung diseases: cystic fibrosis, bronchiectasis, and chronic obstructive pulmonary disease.[J]. Diagnostic Microbiology & Infectious Disease, 2010, 68(1):20–27.	No outcomes of interest
26	Finklea J D, Khan G, Thomas S, et al. Predictors of mortality in hospitalized patients with acute exacerbation of bronchiectasis.[J]. Respiratory Medicine, 2010, 104(6):816–821.	Review
27	Kurahara M, Kokuho N, Hayashi H, et al. Clinical characteristics of COPD with mild bronchiectasis.[J]. European Respiratory Journal, 2011(Suppl 55):3571.	No outcomes of interest

28	O'Donnell A E. Bronchiectasis in patients with COPD: a distinct COPD phenotype?[J]. CHEST Journal, 2011, 140(5): 1107-1108.	editorial
29	Shimizu K, Hasegawa M, Makita H, et al. Comparison of airway remodelling assessed by computed tomography in asthma and COPD.[J]. Respiratory Medicine, 2011, 105(9):1275–1283.	No outcomes of interest
30	Van T N, Wada H, Ogawa E, et al. Recent findings in chronic obstructive pulmonary disease by using quantitative computed tomography.[J]. Respiratory Investigation, 2012, 50(3):78-87.	Review
31	Spyratos D ,, Chloros D ,, Sichletidis L ,. Diagnosis of chronic obstructive pulmonary disease in the primary care setting.[J]. Hippokratia, 2012, 16(1):17-22.	Review
32	Lode H, Allewelt M, Balk S, et al. A prediction model for bacterial etiology in acute exacerbations of COPD.[J]. Infection, 2007, 35(3):143-149.	Did not have control
33	Novosad S A, Barker A F. Chronic obstructive pulmonary disease and bronchiectasis.[J]. Current Opinion in Pulmonary Medicine, 2013, 19(2):133-139.	Review
34	Roca M, Verduri A, Corbetta L, et al. Mechanisms of acute exacerbation of respiratory symptoms in chronic obstructive pulmonary disease[J]. European Journal of Clinical investigation, 2013, 43(5):510-521.	Review
35	Gon calvesa JR, Pereirab MC, De Cerqueiraa EP, et al. Severe obstructive disease: Similarities and differences between smoker and nonsmoker patients with Does coexistence with bronchiectasis influence intensive care unit outcome in patients with chronic obstructive pulmonary disease. *Rev Port Pneumol*.2013;19(1):13–18.	No outcomes of interest
36	J, Rezende Gon?alves, M, Corso Pereira, D, Oliveira Magro, et al. Severe obstructive disease: Similarities and differences between smoker and non-smoker patients with COPD and/or bronchiectasis[J]. Revista Portuguesa De Pneumologia, 2013, 19(1):13–18.	Review
37	Lima A J, Tambascio J, Hc D D S, et al. Transport of mucoid mucus in healthy individuals and patients with chronic obstructive pulmonary disease and bronchiectasis.[J]. Revista Portuguesa De Pneumologia, 2013, 19(5):211-216.	Review
38	Birch J, Johnson G, Jiwa K, et al. S65 Large and small airway epithelial cell senescence present in COPD and bronchiectasis?[J]. Thorax, 2013, 68(Suppl 3):A35-A36.	No outcomes of interest
39	Anwar G A, Nnell M J, Worthy S A, et al. Phenotyping adults with non-cystic fibrosis bronchiectasis: a prospective observational cohort study.[J]. Respiratory Medicine, 2013,	No outcomes of interest

	107(7):1001-1007.	
40	Birch J, Johnson G, Jiwa K, et al. S65 Large and small airway epithelial cell senescence present in COPD and bronchiectasis?[J]. Thorax, 2013, 68(Suppl 3):A35-A36.	No outcomes of interest
41	S-H L, B-C J, Y-M S, et al. Comorbid pulmonary disease and risk of community-acquired pneumonia in COPD patients.[J]. International Journal of Tuberculosis & Lung Disease the Official Journal of the International Union Against Tuberculosis & Lung Disease, 2013, 17(12):1638-1644.	bronchiectasis was confirmed by Chest X-ray
42	Hurst JR, Elborn JS, De Soyza A. COPD-bronchiectasis overlap syndrome.*Eur Respir J*. 2015;45(2):310–313.	comment
43	Miravitlles M, Soler-Cataluña J J, Baranda F, et al. Previous outpatient antibiotic use in patients admitted to hospital for COPD exacerbations: room for improvement[J]. Infection, 2013, 41(2): 361-370.	No outcomes of interest
44	Robert W, Sanjay S, Antonio A, et al. Antibiotics For Treatment And Prevention Of Exacerbations Of Chronic Obstructive Pulmonary Disease.[J]. Journal of Infection, 2013, 67(6):497-515.	Review
45	Soo K S, Joon Beom S, Ho Yun L, et al. Chronic obstructive pulmonary disease: lobe-based visual assessment of volumetric CT by Using standard images--comparison with quantitative CT and pulmonary function test in the COPDGene study.[J]. Radiology, 2013, 266(2):626-635.	Did not have control
46	Ma W, Sheikh K, Svenningsen S, et al. Ultra‐short echo‐time pulmonary MRI: Evaluation and reproducibility in COPD subjects with and without bronchiectasis[J]. Journal of Magnetic Resonance Imaging, 2015, 41(5): 1465-1474.	No outcomes of interest
47	Martínez-Garcia M Á, Selma-Ferrer M J, Soriano C N, et al. Bronchiectasis Phenotype in COPD Patients[J]. Clinical Pulmonary Medicine, 2015, 22(3).	Review
48	Ni Y, Shi G, Yu Y, et al. Clinical characteristics of patients with chronic obstructive pulmonary disease with comorbid bronchiectasis: a systemic review and meta-analysis.[J]. International Journal of Copd, 2015, 10:1465-75.	Review

Fig A. Funnel plots of publication bias for comorbid bronchiectasis and risk for COPD exacerbations. Left side without trim and fill and right with.

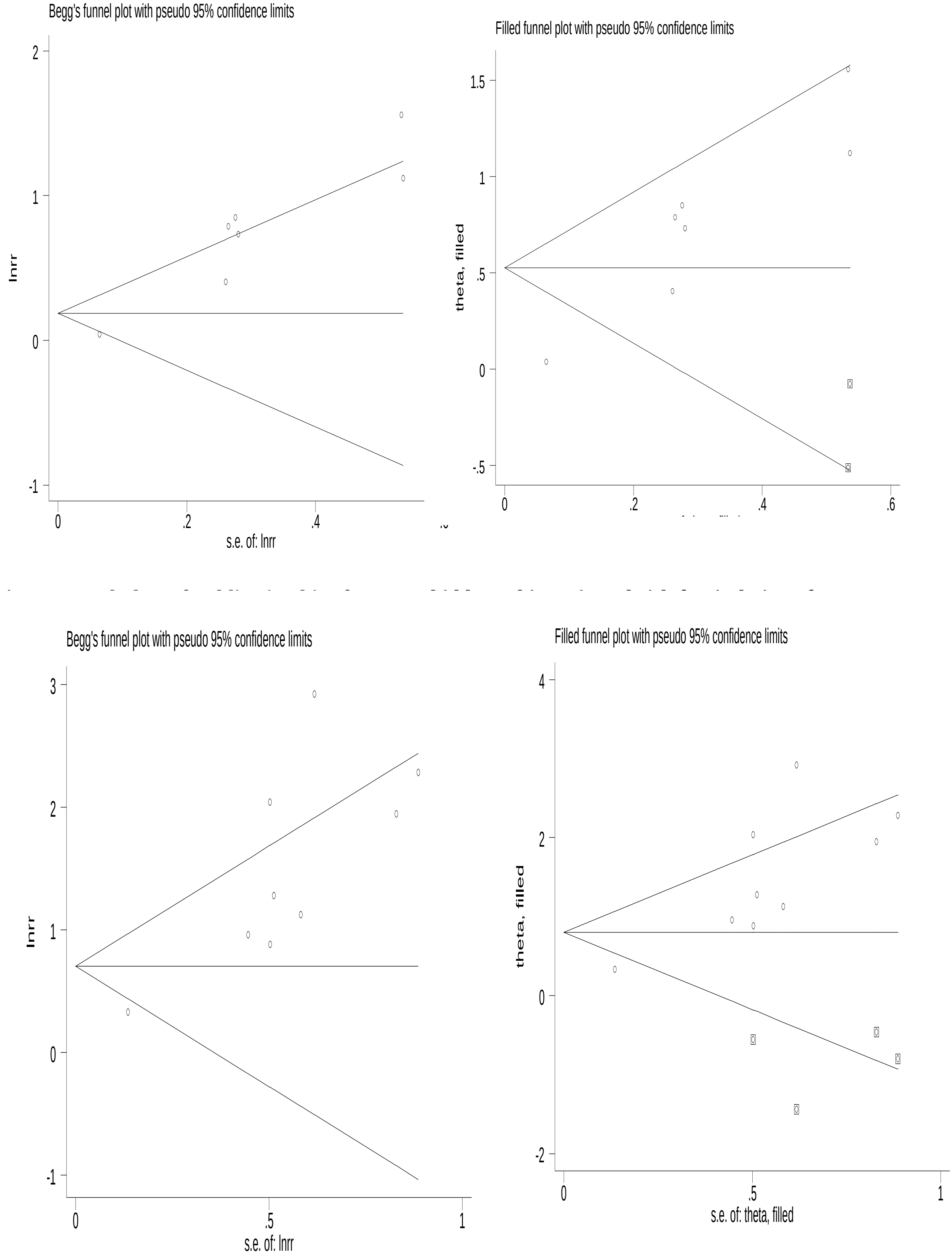

severe obstruction. Left side without trim and fill and right with.

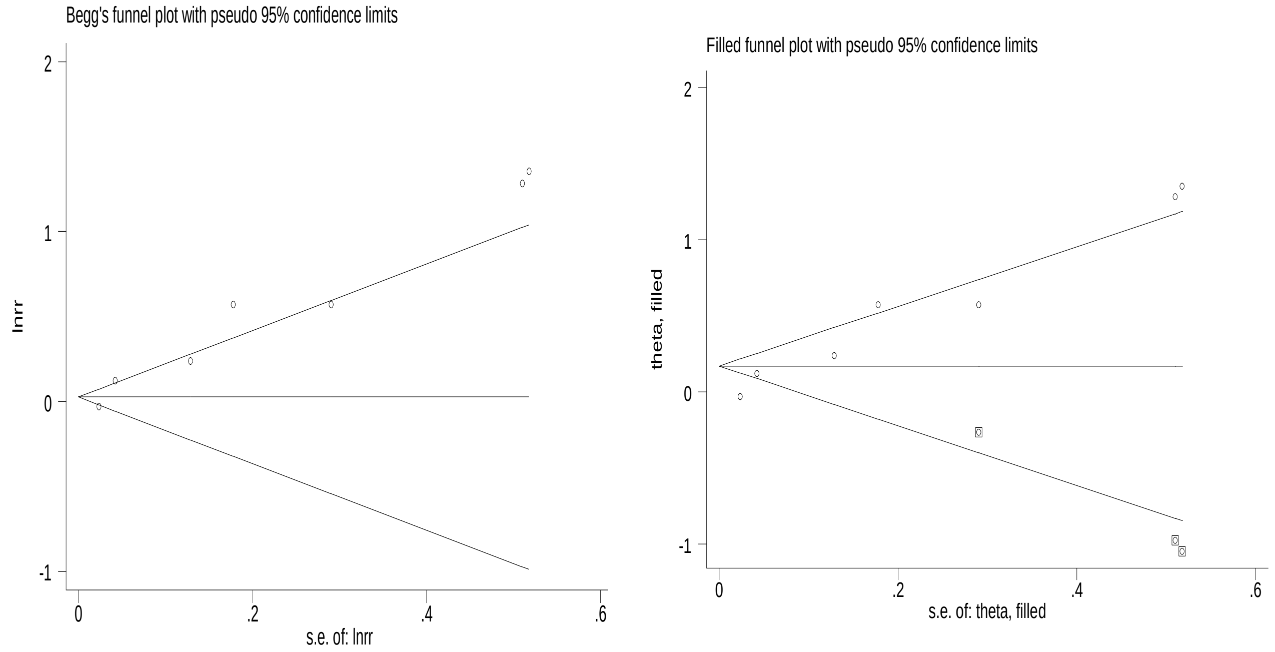

Fig D **Funnel plots of publication bias for comorbid bronchiectasis and risk for mortality.**

Left side without trim and fill and right with.

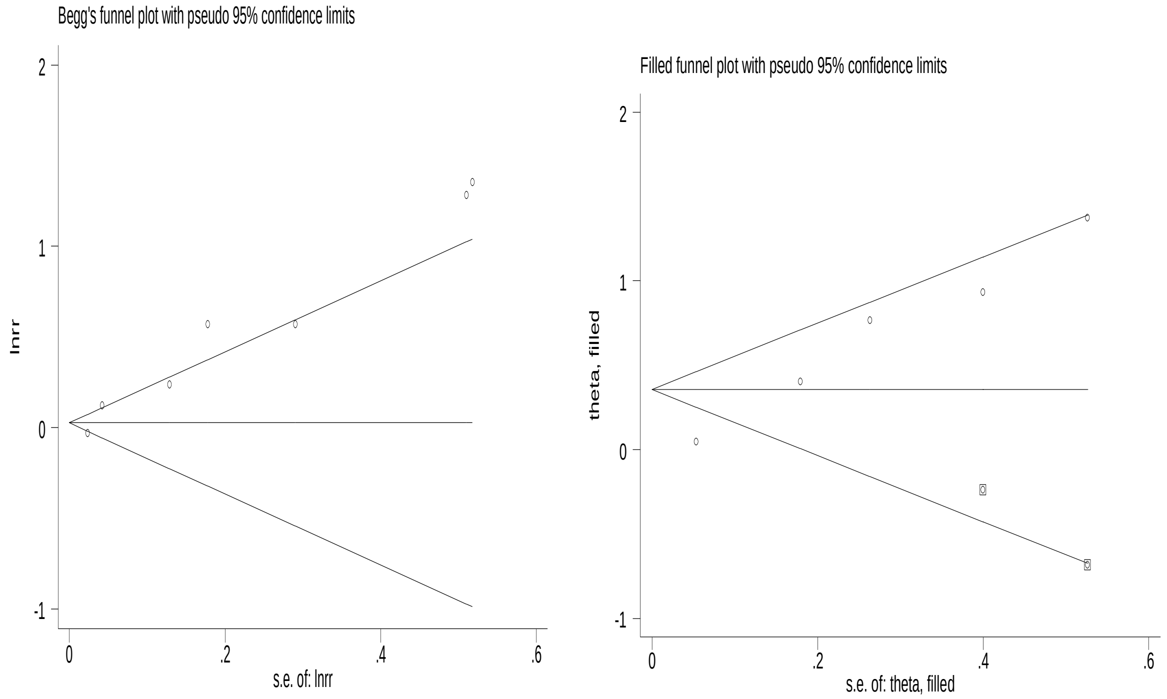

Appendix 1.Commands for each figure

Figure 2, 3, 4, 5.

Stata12.0 Software

```
. gen lnrr=ln(rr)
. gen lnll=ln(ll)
. gen lnul=ln(ul)
metan lnrr lnll lnul, eform label ( namevar=author,yearvar=year) boxsca(0.9)random
```

Figure A, B, C, D.

Stata12.0 Software

```
. gen lnrr=ln(rr)
. gen lnll=ln(ll)
. gen lnul=ln(ul)
gen selnrr=(lnul-lnll)/3.92
metabias lnrr selnrr, graph( begg)
metatrim rr ll ul, ci reffect eform funnel
```
